# A proposed methodology for detecting the malignant potential of pulmonary nodules in sarcoma using computed tomographic imaging and artificial intelligence-based models

**DOI:** 10.3389/fonc.2023.1212526

**Published:** 2023-08-21

**Authors:** Esha Baidya Kayal, Shuvadeep Ganguly, Archana Sasi, Swetambri Sharma, Dheeksha DS, Manish Saini, Krithika Rangarajan, Devasenathipathy Kandasamy, Sameer Bakhshi, Amit Mehndiratta

**Affiliations:** ^1^Centre for Biomedical Engineering, Indian Institute of Technology Delhi, New Delhi, India; ^2^Medical Oncology, Dr. B.R.Ambedkar Institute Rotary Cancer Hospital, All India Institute of Medical Sciences, New Delhi, Delhi, India; ^3^Department of Radiodiagnosis, All India Institute of Medical Sciences, New Delhi, Delhi, India; ^4^Radiodiagnosis, Dr. B.R.Ambedkar Institute Rotary Cancer Hospital, All India Institute of Medical Sciences, New Delhi, Delhi, India; ^5^Department of Biomedical Engineering, All India Institute of Medical Sciences, New Delhi, Delhi, India

**Keywords:** sarcoma, lung metastases, machine learning, deep learning, artificial intelligence, malignancy, radiomics, early diagnosis

## Abstract

The presence of lung metastases in patients with primary malignancies is an important criterion for treatment management and prognostication. Computed tomography (CT) of the chest is the preferred method to detect lung metastasis. However, CT has limited efficacy in differentiating metastatic nodules from benign nodules (e.g., granulomas due to tuberculosis) especially at early stages (<5 mm). There is also a significant subjectivity associated in making this distinction, leading to frequent CT follow-ups and additional radiation exposure along with financial and emotional burden to the patients and family. Even 18F-fluoro-deoxyglucose positron emission technology-computed tomography (18F-FDG PET-CT) is not always confirmatory for this clinical problem. While pathological biopsy is the gold standard to demonstrate malignancy, invasive sampling of small lung nodules is often not clinically feasible. Currently, there is no non-invasive imaging technique that can reliably characterize lung metastases. The lung is one of the favored sites of metastasis in sarcomas. Hence, patients with sarcomas, especially from tuberculosis prevalent developing countries, can provide an ideal platform to develop a model to differentiate lung metastases from benign nodules. To overcome the lack of optimal specificity of CT scan in detecting pulmonary metastasis, a novel artificial intelligence (AI)-based protocol is proposed utilizing a combination of radiological and clinical biomarkers to identify lung nodules and characterize it as benign or metastasis. This protocol includes a retrospective cohort of nearly 2,000–2,250 sample nodules (from at least 450 patients) for training and testing and an ambispective cohort of nearly 500 nodules (from 100 patients; 50 patients each from the retrospective and prospective cohort) for validation. Ground-truth annotation of lung nodules will be performed using an in-house-built segmentation tool. Ground-truth labeling of lung nodules (metastatic/benign) will be performed based on histopathological results or baseline and/or follow-up radiological findings along with clinical outcome of the patient. Optimal methods for data handling and statistical analysis are included to develop a robust protocol for early detection and classification of pulmonary metastasis at baseline and at follow-up and identification of associated potential clinical and radiological markers.

## 1 Introduction

The presence of lung metastases in patients with solid tumors is considered as an important criterion to direct appropriate treatment management and to further prognosticate. Computed tomography (CT) is the standard of care to detect pulmonary metastases and for staging of cancers (especially for intermediate- or high-grade tumors). Indeterminate pulmonary nodules are a very common finding and are often encountered in such clinical scenarios. However, CT is not very appropriate in differentiating metastatic nodules from benign nodules. This issue is even more glaring in developing countries in Asia and Africa where tuberculosis is highly prevalent, and it can be challenging, if not impossible, to differentiate tubercular granuloma from metastatic nodules. The benign and the malignant nodules, especially those at early stages (<5 mm) show very close resemblance to each other and there is a significant subjectiveness involved in making this distinction, requiring frequent follow-up imaging. This leads to increased financial and emotional burden over the healthcare facility and patients as well as unnecessary radiation exposure to patients. Even with the advent of 18F-fluoro-deoxyglucose positron emission technology-computed tomography (18F-FDG PET-CT), while extrapulmonary metastases may be additionally detected (1), the lung nodule conundrum remains a clinical problem. This is because not all metastatic nodules show FDG uptake on PET-CT, and at the same time, active benign nodules such as granulomas might show FDG uptake (1). In addition, small nodules (<5 mm) cannot be reliably evaluated using 18F-FDG PET-CT. Hence, currently there is no non-invasive imaging technique that can reliably characterize the malignant potential of lung nodules.

Lung is one of the favored sites of metastasis in sarcomas and 62%–83% of patients present with lung metastases during their entire disease course, with lung also being the most common site of relapse (2–6). Among patients with sarcomas of extremity, approximately 20% of patients develop isolated pulmonary metastasis at some point in their disease progression (2, 3). In patients who have multiple lung nodules detected with CT, 73% are reported to be metastases (7, 8). Hence, patients with sarcomas, especially those from low- to medium-income countries (LMICs), can provide an ideal platform to develop a model to differentiate metastases from benign nodules in the lung. Various consensus clinical guidelines exist for defining pulmonary nodules as metastatic. The Children Oncology Group definition of evident metastatic disease differs according to sarcoma type and is an area of constant evolution (9), which is left to the discretion of the treating physician (9, 10). For the subcentimeter lesions, optimal management remains unclear (11). The availability of thin slice CT technology introduced further uncertainty by detecting nodules <5 mm in diameter, thereby increasing the frequency of positive tests (12). There is no accurate non-invasive modality to determine the malignant potential of such smaller pulmonary nodules, and invasive biopsy of these small nodules is often not feasible. Moreover, in daily practice, overlapping radiologic features of metastases are frequently encountered, which makes distinction from other non-malignant pulmonary diseases difficult. For example, osteosarcoma metastases may appear as benign calcified pulmonary nodules, though as many as 40% of osteosarcoma lesions are not calcified and unusual forms of metastasis are also observed (7, 13).

To overcome the lack of optimal specificity of CT scan in detecting metastasis, it is imperative to develop a non-invasive imaging-based computational model to differentiate pulmonary nodules between benign and malignant etiologies. A lot of work has been reported for the detection and classification of primary lung cancer using machine learning (ML) and deep learning (DL) (14–20); however, predicting the malignant potential of the lung nodules in clinical scenarios of possible metastases from solid tumors is still an unmet need. Therefore, in this protocol study, we are proposing to utilize ML/DL techniques to identify lung nodules and develop a prediction model to characterize them as benign nodules or malignant lung metastases. The objectives of the proposed protocol are as follows: (a) identification of clinical and radiological markers for differentiation of lung metastases from benign lung nodules; (b) training and testing of classification model for lung metastases using retrospective training cohort; and (c) clinical validation of the developed classification model using both retrospective and prospective validation cohort.

## 2 Methods and analysis

### 2.1 Literature review on application of machine learning- and deep learning-based techniques for lung nodule characterization

Numerous studies have been reported for classification of primary lung cancer and benign lung nodules using ML/DL techniques (14–20). Extraction of effective discriminative features for lung nodule characterization is challenging due to complex anatomical structures in thoracic CT images. Many reported methods have shown improved classification performance toward this direction, and strategically, these methods can be categorized into two groups: traditional ML-based methods using feature engineering and DL-based methods.

The former methods employ feature engineering to extract various handcrafted features, like size, shape, margin, intensity, and statistical textural features in ROIs; next, identify effective discriminating features applying feature selection strategies (21, 22) and finally develop a classification model using ML algorithms, viz., logistic regression (LR), support vector machine (SVM), K-nearest neighbors (KNN), random forest (RF), linear discriminant analysis (LDA), deep neural network (DNN), and Adaboost (14–17). Various shape, margin, intensity, and textural features using GLCM in 2D axial plane and 3D volume of lung nodule (23–28), textural features using phylogenetic diversity (25), and wavelets (29, 30) were used to classify malignant and benign lung nodules using SVM, LDA, DNN, and naïve Bayes classifiers. Textural features using local binary pattern (LBP) and discrete cosine transform were extracted and used to train SVM and KNN models for lung nodule characterization (31). Shape, intensity, statistical textural features using GLCM, Gabor filters, and LBP were used to train RF models to classify lung nodules as malignant and benign (32, 33), and SVM, KNN, and LR models were used to classify different nodule types like solid, semi-solid, and ground-glass, respectively. A comparative study evaluating various ML models showed that an ensemble classifier combining SVM and RF produced the best classification performance for malignant nodules compared to KNN, LDA, and AdaBoost using shape, size, and texture-based features (34). Similarly, morphological and statistical features were applied to an ensemble of three classifiers utilizing multilayer perceptron (MLP), KNN, and SVM to classify benign or malignant lung nodules (35).

While handcrafted features need expert domain knowledge for pulmonary CT images, DL does not require explicit features extraction; it reveals intrinsic structural properties in input data by applying brain-inspired computing and has showed notable improvement in medical image analysis (18–20). Various frameworks based on convolutional neural networks (CNNs) have been developed for lung nodule detection and an elaborated review can be found in Refs (36–39). Applying reinforcement learning, region proposal network (40), faster region-based CNN (41), various advanced CNN models (42–45), and ensemble learners using multiple CNN models (46) have been designed for lung nodule detection and false-positive reduction. CNN-based dense convolutional binary-tree networks (47) and spatial pyramid dilated network (48) were developed to derive useful features from image data to discriminate malignant pulmonary nodules from benign nodules. Optimal Ensemble framework combining multiple CNN models using ResNet, AlexNet, DenceNet, InceptionNet, and SqueezeNet (49–53), transfer learning-based system (54–57), and hybrid CNN-based system (58–62) have been reported for classifying malignant and benign lung nodules. A research group proposed multi-view collaborative deep CNN models for incorporating prior knowledge about the association of nodule’s malignancy and its heterogeneity (49) and further showed learning from ambiguous labels for more accurate lung nodule malignancy prediction (51). 3D Deep CNN and SVM with multiple kernel learning algorithms was proposed to fuse the DL features with clinical information for lung nodule diagnosis (61). 3D CNN and RF were used to combine CT imagery with biomarker annotation and volumetric radiomics features for lung nodule malignancy prediction (62). CNN with adaptive morphology and textural features (63), 3D segmentation attention network-based systems integrating asymmetric convolution with a gradient boosting machine (64), and 3D non-local network-based systems incorporating channel attention and adaptive network growth algorithm (65) were reported for lung nodule classification. Lung nodule classification was performed using features learned from two deep 3D customized mixed link network with gradient boosting machine (66). Studies have shown improved classification accuracy for malignant nodules using optimal deep feature selection from different CNN-based convolution layers and fusion of the deep features for the final classifier (67–69). A multi-scale cost-sensitive neural network was proposed to mitigate the issue of insufficient labeled data and class imbalance (70). A soft activation mapping-based method meta-learning scheme was reported for interpretable lung nodule classification (71) and a meta ordinal set was further generated by the same research group by developing meta ordinal weighting network to explore the ordinal relationship between the data for lung nodule classification (72). Recently, DL models based on transformers (73, 74) or combined with CNN and transformers (75, 76) have been successfully applied for lung nodule detection and classification. A self-supervised region-based 3D transformer model was developed to identify lung nodules among a set of candidate regions (73). A local focus scheme was incorporated into a deformable dilated transformer to develop a multi-granularity dilated transformer to focus on the more discriminative local features to classify lung nodules in CT scans (74). TransUnet was developed based on the transformer to encode feature representations of input CT scans and the Unet network to decode the hidden feature for outputting the final classification results (75). Res-Trans was developed using convolutional operations to extract local features and transformer blocks with self-attention to capture global features (76).

Few research gaps have been identified from the above literature survey for lung nodule characterization. Firstly, all the studies reported development of detection and classification models for primary lung cancer nodules, while characterizing metastatic lung nodules, which are also prevalent among patients with various primary cancers, have not or rarely been explored. Secondly, it has been observed that clinical features, which are also informative for lung nodule characterization (6, 77, 78), have not been utilized by most of the reported ML and DL methods. Thirdly, temporal changes in lung nodules during treatment, which may be captured by radiological and/or radiomics features from follow-up CT scans and might be useful for predicting malignancy at early stage, have not been considered by the earlier studies. Fourthly, most of the studies have used publicly available retrospective thoracic CT datasets for training and validation of the proposed models; however, prospective datasets may be better suited to test the generalizability of the model’s performance. These issues have been addressed in the current study protocol.

### 2.2 Study design

It is an ambispective cohort study with a retrospective training cohort and a prospective validation cohort. Patients with bone and soft tissue sarcomas who registered for treatment between January 2011 and December 2023 in the Medical Oncology Clinic of Dr. B.R.Ambedkar Institutional Rotary Cancer Hospital (IRCH), All India Institute of Medical Sciences (AIIMS) New Delhi, India will be included in this study. CT scans at baseline and follow-up with the presence of lung nodules and clinical data available in institutional databases will be used as training and testing datasets for the proposed classification model. For the validation cohort, patients with bone sarcomas and soft tissue sarcomas will be prospectively recruited from the Medical Oncology Clinic of Dr. B.R.A. IRCH, AIIMS New Delhi, India and these prospectively performed CT scans and prospectively collected clinical data will be used for the validation of the proposed classification model.

### 2.3 Sample size

For a two-class classification problem, considering a group ratio of 1:1, to achieve an area under the receiver operating characteristics curve of 0.8 with a 20% absolute error margin in a two-sided 95% confidence interval, a minimum of 34 samples will need to be investigated. However, for supervised learning models, studies have shown that increasing sample size beyond 1,000/class demonstrated no further significant improvements in the overall classification accuracy (79). For any patient, each annotated nodule in the lung will serve as a sample in the training/testing process during model development. In the proposed model, CT scans of a minimum of 500 patients will be used retrospectively, and considering an average of 5 nodules (range: 1–10) per CT scan, a minimum of ~2,500 samples of lung nodules will be annotated. For validation of the proposed model, CT datasets of a minimum of 50 patients will be prospectively collected. For the development of the proposed prediction model, retrospective CT datasets will be analyzed, and for validation of the proposed model, both retrospective and prospective CT datasets will be analyzed. A total of 2,000–2,250 sample nodules from at least 450 patients from the retrospective cohort will be used for training and testing the prediction model for characterizing lung metastases of sarcoma. Nearly 250 nodules from 50 patients from the retrospective cohort and 250 sample nodules from 50 prospective cohorts comprising a total of 500 nodules will be used exclusively for validating the proposed prediction model.

### 2.4 Data collection

Patients with bone and soft tissue sarcomas who registered for treatment during January 2011 to December 2023 in the outpatient department (OPD) of Dr. B.R.Ambedkar IRCH, AIIMS New Delhi, India will be considered for inclusion in this project.

Inclusion criteria for the retrospective cohort (registered during January 2011–December 2021) will be as follows: (a) patients with biopsy-proven bone or soft tissue sarcoma and (b) having lung nodule(s) in the chest CT scan at the time of presentation or in the course of treatment. Exclusion criteria for the retrospective cohort will be as follows: (a) patients who could not be adequately followed up and decision about lung nodule(s)’s malignancy could not be reached and (b) patients did not undergo chest CT scan in the course of treatment.

For the prospective cohort (registered during January 2022–December 2023), inclusion criteria will be as follows: (a) patients with biopsy-proven bone or soft tissue sarcoma and (b) having lung nodule(s) in the chest CT scan at the time of presentation. Exclusion criteria for the prospective cohort will be as follows: (a) patient did not undergo CT scan in the course of treatment, (b) patients whose CT scan could not be retrieved, (c) refusal for informed consent, and (d) patient for whom decision about the nature of the lung nodule(s) could not be reached within the follow-up time. For the prospective cohort, follow-up time will be at least 1 year or till dropout due to death or other reason, whichever is earlier.

For the development of the proposed classification model, a retrospective dataset of patients who registered during 2011–2018 will be used. For validation, a retrospective dataset of patients who registered during 2019–2021 and a prospective dataset of patients who will be recruited during 2022–2023 in the Medical Oncology Clinic of Dr. B.R.Ambedkar IRCH, AIIMS New Delhi, India will be used.

### 2.5 Ground-truth annotation and labeling of lung nodules

#### 2.5.1 Ground-truth region of interest for lung nodule

Conventionally, the demarcation of ground-truth region of interest (ROI) for lung nodules is performed by an expert radiologist by freehand manual drawing of ROI on CT scan using annotation software after thoroughly scanning the whole CT slices. As manual annotation tasks are very tedious and time-consuming for radiologists and due to severe workload and increased number of scans, radiologists’ decision-making may suffer from human error and inter- and intra-observer subjectivity. To mitigate these challenges, a semiautomated segmentation of lung nodules requiring only a minimal human input has been developed in-house at the Center for Biomedical Engineering, IIT Delhi, India. Using the developed semi-automated segmentation tool, demarcation of ground-truth ROI for lung nodules has been initiated under the Department of Radiodiagnosis, AIIMS, Delhi, India.

**Figure 1:**
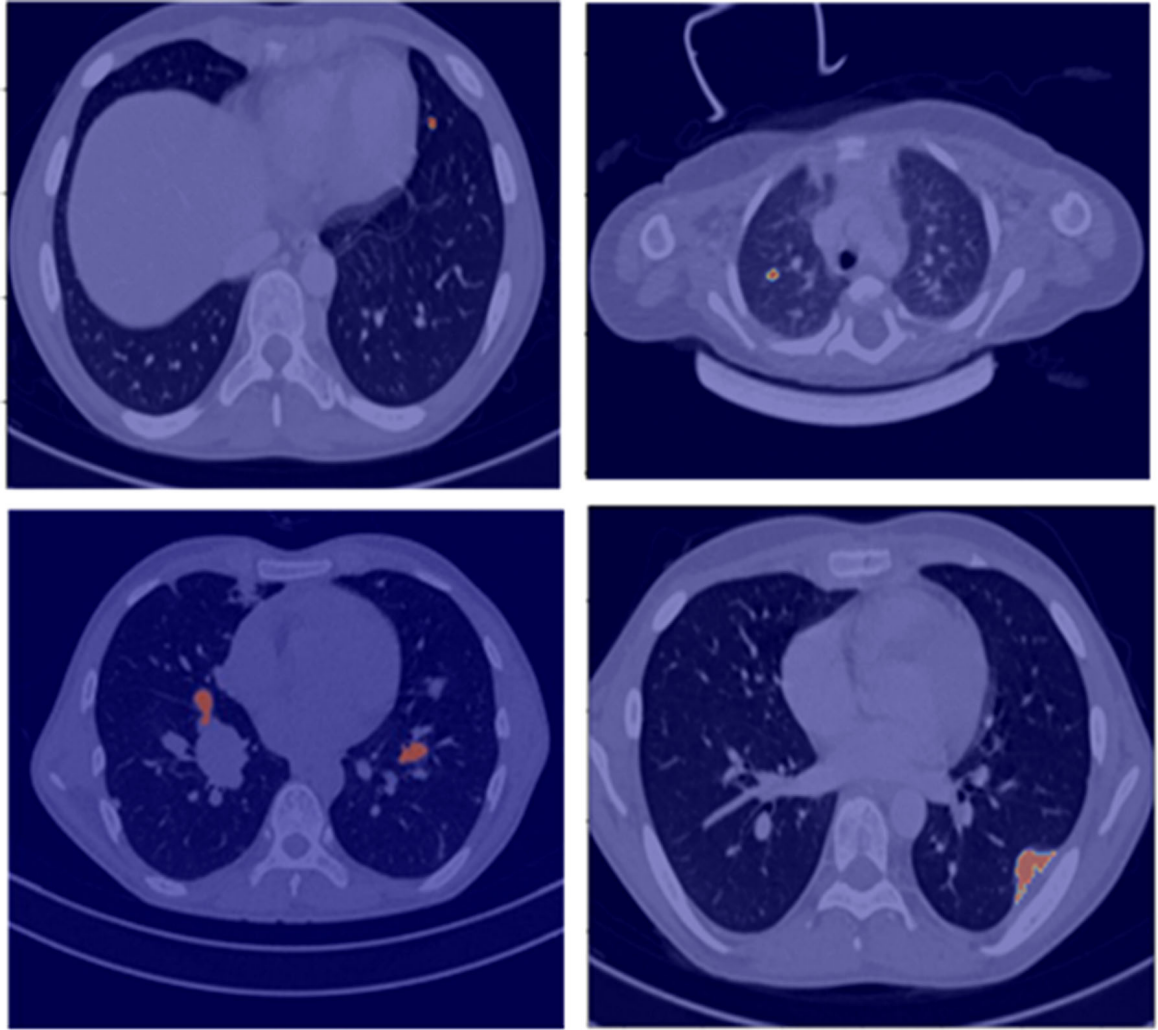
Segmentation results (red overlay) of lung nodules for a representative patient (25 years, male) with sarcoma.

The developed lung nodule segmentation algorithm is based on the region-growing method and morphological image processing algorithms. It requires only manual input for selecting a seed-point inside the nodule on a CT image and then performs automatic segmentation of the nodule across the slices of the CT image. The algorithm is capable of automatically segmenting out the lung nodules having different shapes, sizes, locations, and characteristics like solitary, juxta-pleural, ground-glass, and juxta-vascular nodules and has shown promising results in limited datasets of 50 patients for initial assessment. This segmentation tool will be used by the radiologists for ground-truth annotation of lung nodules. Examples of segmentation results for lung nodules using the developed algorithm are shown in **Figure 1**. This segmentation will be verified by two expert radiologists with 12 and 20 years of experience, respectively. Any disagreements will be resolved by mutual discussion to build a consensus.

#### 2.5.2 Ground-truth labeling of lung nodule as metastatic or benign

**Figure 2:**
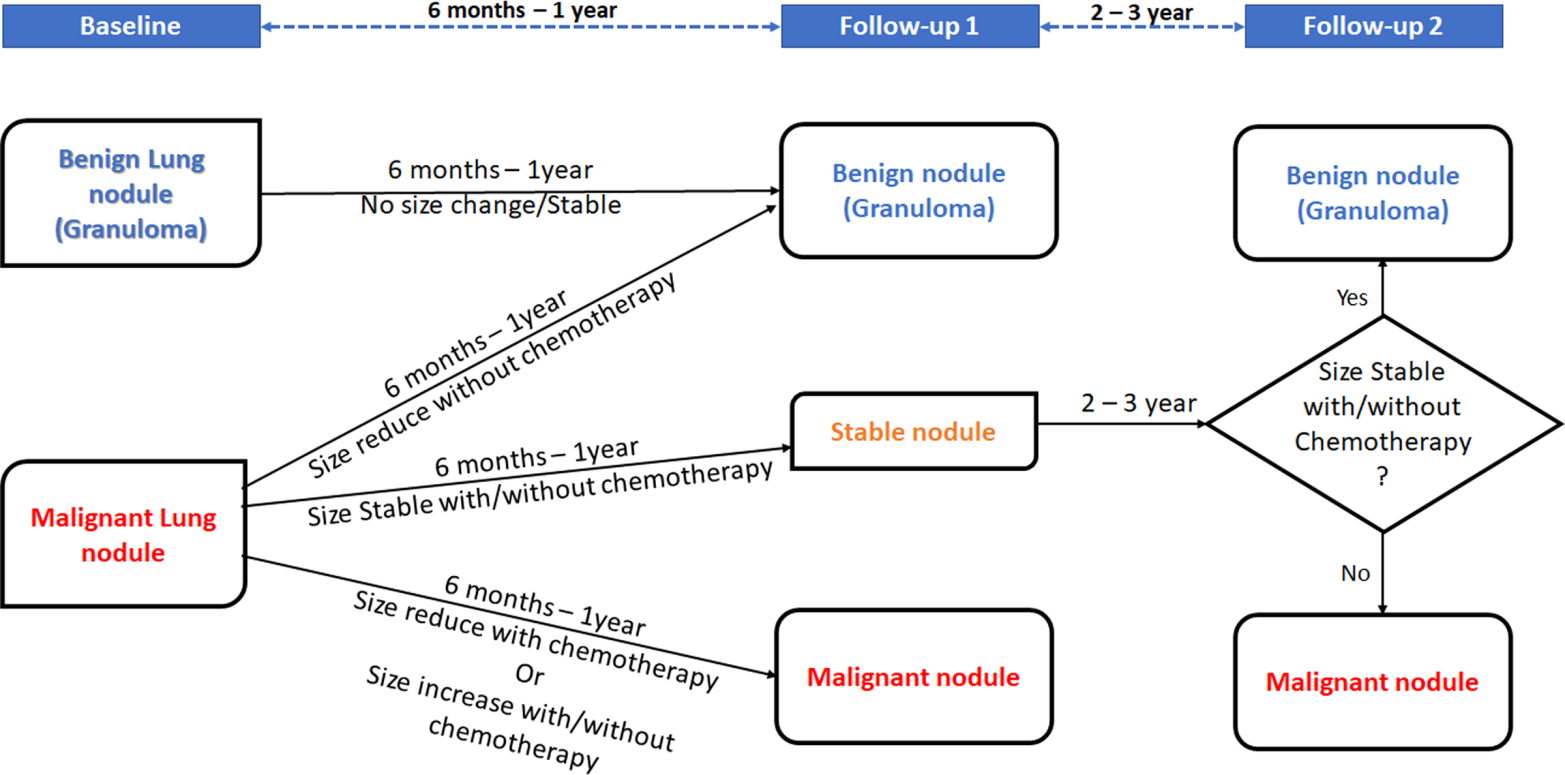
Decision rule for malignant lung nodules depending on patient outcome and/or radiological changes.

Ground-truth labeling of lung nodules as metastatic or benign in CT scans will be performed based on a biopsy report if available or consensus decision depending on the clinical outcome of the patient and/or radiological changes in follow-up scans as summarized in **Figure 2**. At baseline imaging (prior to administration of chemotherapy), if consensus opinion at baseline suggests that the nodule can be clearly labeled as benign (no known malignancy, presence of calcification within the nodule in a patient with non-osteogenic sarcoma, history of tuberculosis, and presence of other features of tuberculosis), it will be labeled as benign. The label assigned at benign will be revised on the basis of follow-up imaging. After 6–12 months, if a lung nodule designated as benign at baseline remains stable, then it is assigned a definitive label of a benign lung nodule. On the other hand, if a lung nodule designated as malignant at baseline shows a size reduction after 6–12 months without undergoing chemotherapy, it is also assigned a definitive label of a benign lung nodule. If a malignant lung nodule at baseline shows a size reduction after chemotherapy or shows a size increase with or without chemotherapy, it is assigned a label of malignant lung nodule after 6–12 months. In contrast, if a malignant nodule at baseline remains stable during the next 6–12 months with or without chemotherapy, it is considered as a stable nodule and observed over the next 2–3 years for any change in size. If the size of the stable nodule continues to be stable over this time with or without chemotherapy, it is concluded as a benign nodule, likely granuloma. If the size of the stable nodule changes after 2–3 years whether being treated with chemotherapy or not, it is concluded as malignant.

### 2.6 Workflow

**Figure 3:**
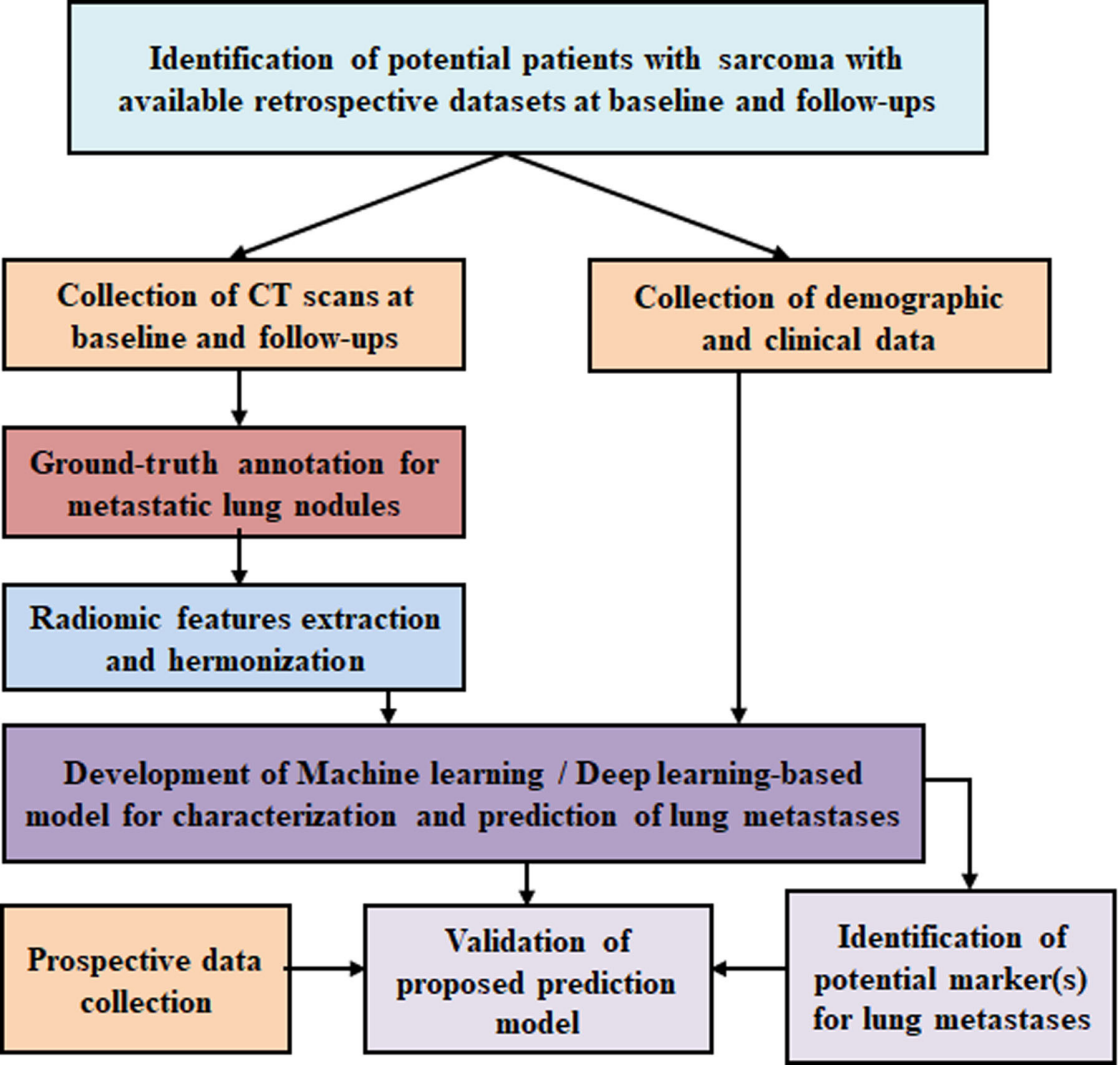
Flowchart of the proposed study protocol.

The proposed study is composed of the following steps and summarized in the flowchart in **Figure 3**:

**a) Retrospective data collection**

Screening of available retrospective CT scans and clinical data of soft tissue sarcoma and bone sarcoma patients with known clinical follow-up details registered at Dr. B.R.Ambedkar IRCH, AIIMS New Delhi, India, between January 2011 and December 2021 will be performed. We anticipate that datasets of at least 500 patients will be collected.

**b) Prospective data collection**

For validation of the proposed model, CT scans and clinical data of nearly 100 patients will be prospectively collected at Dr. B.R.Ambedkar IRCH, AIIMS New Delhi, India between January 2022 and December 2023.

**c) Ground-truth preparation**

Ground- truth annotation and collation of all individual CT scans at baseline and follow-ups will be performed using the in-house semi-automated lung nodule segmentation tool (**Figure 1**). Ground-truth labeling of lung nodules as metastatic or benign will be performed using histopathological results of biopsy or metastatectomy (if available) or will be based on multi-disciplinary opinion based on baseline and/or follow-up radiological findings along with the clinical outcome of the patient (**Figure 2**).

**d) Radiological feature extraction**

Radiological characteristics of lung nodules will be reviewed on CT scans at baseline and follow-up and relevant features like (i) nodule size, (ii) nodule position, (iii) change of size during follow-up, (iv) types of calcifications, (v) air bronchogram, (vi) surrounding ground-glass opacity, (vii) surrounding fibrosis, (viii) no feeding vessel sign, and (ix) bilaterality will be captured and analyzed.

Extraction of various radiomics features from the annotated ground-truth lung nodules will be performed to train the proposed classification model for identification of lung metastasis. The radiomics features consist of the following: (i) shape-based features, (ii) first-order textural features from histogram, (iii) second-order textural features from gray-level cooccurrence matrix (GLCM), and (iv) higher-order textural features from neighborhood gray-tone difference matrix (NGTDM) and run length matrix (RLM) will be evaluated. A detailed tentative list of radiomics features is listed in **Table 1**. Studies have shown that handcrafted features from tissues surrounding the lung nodule provide global representation of nodule CT images and can be informative for lung nodule characterization (80, 81). Therefore, the radiomics features in lung parenchyma surrounding the lung nodule will also be evaluated and analyzed. Temporal changes in semantic radiological and radiomics features in lung nodules will be evaluated from follow-up CT images (if available) during treatment and will be used along with baseline features. CT data are intrinsically dependent on the protocol acquisition parameters and pixel values in CT data are directly related to the physical characteristic of the tissues having different linear attenuation coefficients. Therefore, harmonization processes will be applied on extracted radiomics features to make them independent of scanner-specific parameter dependencies.

**Table 1:** List of radiomics features to be evaluated for lesion identification, characterization, diagnosis, monitoring prognosis or predicting outcome of patient.

SN	Radiomics Features		Description if any for easy understanding or describing importance of feature
	Methods	Features	
1	**Shape**	Volume	Volume in 3D
2		Eccentricity	Measure of aspect ratio, which is the ratio of the length of the major axis to the length of minor axis.
3		Axis of least inertia	It is defined as the line for which the integral of the square of the distances to points on the shape boundary is a minimum. It is unique to the shape.
4		Euler number	The difference between the number of contiguous parts and the number of holes on a shape.
5		Minimum bounding rectangle	Smallest rectangle that contains every point in the shape.
6		Circularity ratio	The ratio of the area of a shape to the area of a circle having the same perimeter.
7		Convexity	The ratio of perimeters of the convex hull over that of the original contour.
8		Solidity	The extent to which the shape is convex or concave.
9	**Histogram**	Mean	Average intensity of image
10		Variance	Contrast in intensity levels present in the image
11		Skewness	Measure of the asymmetry of histogram with respect to mean
12		Kurtosis	Measure of relative flatness and shape of histogram
13		Energy	Measure of uniformity; maximum value is 1 for constant image
14		Entropy	Measure of variability; minimum value is 0, for constant image
15	**Gray-Level Cooccurrence Matrix (GLCM)**	Energy	Measure of image homogeneity
16		Contrast	Measure of local image variation
17		Entropy	Measure of randomness of gray levels
18		Homogeneity	Measure of local homogeneity
19		Correlation	Measure of image linearity
20		Sum average	Measure of overall image brightness
21		Variance	Measure of spread of gray-level distribution
22		Dissimilarity	Measure the coarseness in image
23		Autocorrelation	Measure the repetitive nature of the texture primitive
24	**Neighborhood gray-tone difference matrix (NGTDM)**	Coarseness	Measure spatial rate of change in intensity, thus measure the level of coarseness.
25		Contrast	Measure intensity difference between neighboring regions
26		Busyness	Measure the rapid changes of intensity from one pixel to its neighboring regions
27		Complexity	Measure the rapid spatial changes in intensity
28		Strength	Measure the boldness or distinctiveness of the texture primitives
29	**Gray-level run length method (RLM)**	Short Run Emphasis (SRE)	Measures the distribution of short runs and is expected large for fine textures
30		Long Run Emphasis (LRE)	Measures distribution of long runs and is expected large for coarse structural textures
31		Gray-Level Nonuniformity (GLN)	Measure the gray-level nonuniformity
32		Run Length Nonuniformity (RLN)	Measure the nonuniformity of the length of runs
33		Run Percentage (RP)	Measures the homogeneity and the distribution of runs of an image in a specific direction.
34		Low Gray-Level Run Emphasis (LGRE)	Measures the distribution of low gray-level values
35		High Gray-Level Run Emphasis (HGRE)	Measures the distribution of high gray-level values
36		Short Run Low Gray-Level Emphasis (SRLGE)	Measures the joint distribution of short runs and low gray-level values.
37		Short Run High Gray-Level Emphasis (SRHGE)	Measures the joint distribution of short runs and high gray-level values.
38		Long Run Low Gray-Level Emphasis (LRLGE)	Measures the joint distribution of long runs and low gray-level values.
39		Long Run High Gray-Level Emphasis (LRHGE)	Measures the joint distribution of long runs and high gray-level values.

**e) Clinical features**

The historical records of the eligible patient cohort will be reviewed, and all the relevant clinical details including demographic profile, histological variants, treatment details, and disease outcomes will be captured and analyzed. Data pertaining to the following clinical characteristics and laboratory parameters at baseline will be included: (i) age at the time of diagnosis, (ii) gender, (iii) histologic subtype of sarcoma (osteosarcoma, primitive neuroectodermal tumor, or soft tissue sarcoma), (iv) histological grade, (v) site of primary disease, (vi) the presence of metastases to other sites, (vii) symptom duration prior to presentation, (viii) performance status, (ix) hemoglobin, (x) total leucocyte count (TLC), (xi) platelet count, (xii) serum lactate dehydrogenase (LDH), (xiii) serum alkaline phosphatase (ALP), (xiv) serum C-reactive protein (CRP), and (xv) serum albumin. The baseline factors may aid in estimation of the disease burden and the likelihood of lung metastases. In addition, details pertaining to chemotherapy and local site therapy modalities received (surgery and/or radiotherapy) including the timeline of receipt, therapeutic responses, and event-free survival (EFS) and overall survival (OS) will be collected. The radiological changes in the lung nodules will be assessed in the context of the timeline of therapy received and clinical responses to therapy. The degree of clinician suspicion for lung metastases based on retrospective file review will be mentioned as “high”, “unclear”, or “low”. Clinical high suspicion of metastases is based on the progressive size of lung nodules in patients along with documented clinical deterioration or death or change of line of therapy. The degree of radiologist suspicion based on routine radiological findings will be mentioned separately by the radiologist. If there is a discrepancy between the two, a multidisciplinary discussion between the radiologist and the clinician will be carried out to reach a consensus. The ground truth as to whether the patient has lung metastases or not on imaging will be mentioned subsequently. For this study, OS will be defined as the time from treatment initiation till death from any cause. EFS will be defined as the time from treatment initiation till disease relapse/progression or death from any cause.

**f) Feature selection**

Dimensionality and redundancy reduction and identification of potential radiological and clinical feature(s) will be performed for training the proposed model. Feature selection methods like Fisher’s score, Lasso regularization, RF importance, and Recursive feature elimination will be applied on radiological and clinical feature sets separately for evaluating the importance of the features for the classification task and the final feature selection will be performed as the requirement of the training algorithm.

**g) Machine learning/deep learning models for detection and classification of metastatic lung nodule**

A number of studies have been reported for the classification of benign and malignant lung nodules using ML/DL techniques in primary lung cancer (14–20); however, classification of metastatic lung nodule using artificial intelligence (AI) has been rarely addressed. Various ML-based classification models for primary lung cancer have been reported, while SVM (23–25, 27, 29, 31, 34, 82) was applied mostly along with the other ML technologies like LR (82), KNN (31, 35, 82), RF (32, 33), LDA (27, 34), DNN (28, 30), and Adaboost (34). DL-based frameworks based on CNNs have been developed for lung nodule detection (36). Various advanced CNN models (41–45) and ensemble learners using multiple CNN models (46, 49–53), transfer learning-based systems (54–57), and hybrid CNN-based systems (58–62) have been reported for classifying malignant and benign lung nodules. CNN with adaptive morphology and textural features (63), deep feature selection from different convolution layers (67–69), and 3D segmentation attention network-based systems (64, 65) were reported for lung nodule classification. Recently, DL models based on transformers (73, 74) along with CNN (75, 76) have reported promising results for lung nodule detection and classification.

The plan in the current protocol is to train the proposed model using CT datasets of nearly 450 patients from a retrospective cohort. The aim is to detect and classify the metastatic lung nodules at baseline, i.e., even before commencement of treatment and at follow-up. A separate analysis will be carried out to train the classification model to predict the metastasis even for the smaller nodule (<5 mm) at its very early stage. For the classification model, ML-based algorithms like (i) multivariate LR, (ii) SVM, (iii) LDA, and (iv) RF and DL-based frameworks like (i) transformers with CNN, (ii) 3D CNN (CNN), (iii) recurrent CNN, and (iv) Spatial pyramid Pooling CNNs will be tested and validated. After features selection, relevant radiological features and relevant clinical features will be used separately and in combination to train the ML-based algorithms. For each ML algorithm, three separate training models will be prepared using (i) selected radiological features, (ii) selected clinical features, and (iii) selected radiological and clinical features in combination. For ML model training data normalization, data noise reduction and regularization techniques like ridge regularization and lasso regularization with k-fold cross-validation will be applied as applicable for the model to avoid overfitting and maintain generalizability of the model. The prediction accuracies of different training models for all ML-based algorithms will be compared to identify the best-performing prediction model. For DL-based models, convolutional layers will be used to extract the features from the labeled CT images, then the clinical features will be concatenated with the extracted features vectors from convolutional layers, which will be further feed to the fully connected layers to train the prediction model. Appropriate hyper-parameters like learning rate, dropout, batch size, loss function, momentum, and optimizer will be applied during DL model training. Data augmentation is an important step to overcome insufficiency of labeled data, prevent overfitting, and increase the training accuracy of a DL model. Many literatures reported that use of geometric transformations (55, 60, 65, 76, 83), kernel filtering, color and noise augmentation (50), and Generative Adversarial Networks (84, 85) for augmenting pulmonary CT data improved the accuracy for lung nodule characterization. Data augmentation may introduce inconsistency among data distribution between training and test data. To refine the trained model and maintain the model accuracy, the DL model will be trained on augmented data first, followed by data without any data augmentation. Prediction performance of applied DL-based techniques will be compared to determine the optimal prediction model balancing computational cost and accuracy. For ML-based model implementation, MATLAB^®^ (MathWorks Inc., v2018, Philadelphia, USA) and Python version 3.9 (Python Software Foundation, https://www.python.org/) will be used, and for DL-based model development, programming environments PyTorch (https://pytorch.org/), TensorFlow (https://www.tensorflow.org/), and Keras (https://keras.io/) will be used.

**h) Identification of potential marker(s) for lung metastases**

The proposed model will be able to identify the potential clinical and/or radiological marker(s) for classifying the metastatic lung nodules from benign ones. The proposed prediction model for detecting lung metastases will be developed and tested for clinical and radiological marker(s) separately and in combination, and the importance of both in combination will be determined. This will help to improve the model performance, reducing the misdiagnosis and false-positive results particularly for the early-stage smaller metastatic lung nodules.

**i) Validation**

The validation of the proposed ML/DL-based prediction model will be performed using retrospective as well as prospective clinical and CT datasets of a total of 100 patients, 50 patients each from the retrospective and prospective cohort.

### 2.7 Statistical analysis

a) Student’s *t*-test will be used for continuous variables and the Chi-square test will be used for categorical variables. A *p*-value of <0.05 will be taken as significant.

b) Dimensionality reduction and identification of unique features to train the classification model for lung metastasis detection.

c) ML methods to identify independent radiological and clinical feature(s) as markers of lung metastases.

d) ROC curve analysis will be used to find which feature or combination of features would best classify the lung metastases from the other existing benign lung nodules.

e) True-negative and true-positive rate, positive predictive value or precision, recall, F score, and average false-positive rate per patient will be used to evaluate the performance of the proposed models.

Data analysis and development of an ML-based classification model for lung metastases detection will be performed at the laboratory facility of the Centre for Biomedical Engineering, IIT Delhi, India.

## 3 Discussion

The identification of metastatic disease at presentation is vital to the clinician since the expected prognosis and treatment outcomes are different in metastatic disease from that in localized disease. Furthermore, it allows the clinician to decide the intent of treatment with greater clarity. Thus, determining the nature of lung nodules in a clinical scenario of possible pulmonary metastases is of great importance.

Out of all the medical imaging techniques, CT is considered to be one of the most effective means of detecting lung cancer early (86, 87). Clinicians need to diagnose malignant nodules accurately by reading the patient’s lung CT image; however, reading a large number of CT images is not only time-consuming, and there is also a high probability of misdiagnosis. There has been a lot of research work done regarding the differentiation between benign and malignant solid lung nodules. Lung nodules can be evaluated according to diameter, area, or volume. Results from the literature agreed that volume measurement is a method with a better performance in nodule sizing, as well as in assessing the nodule’s growth (88). Mehta et al. (89) added volumetric nodule measurement to an existing prediction model for malignancy estimation of nodules, showing an improvement in the number of nodule classification. There are a number of other factors like tumor image intensity, shape, and texture that help in determining probability of malignancy in lung nodules (90–97). When evaluating individuals with lung nodules, the probability of malignancy is estimated on the basis of patient-related clinical factors like primary tumor grade, tumor size, and histology type (6, 78) and nodule characteristics, including size (77). As regards morphological characteristics of nodules, besides small size, diffuse, central, laminated, or popcorn calcifications, fat tissue density and perifissural location have been recognized as indicative of benign lesions. It has been found that pleural tags and contour may be identified as independent predictors of pulmonary metastases (98) or higher mean attenuation and larger diameter are significant predictors for pulmonary metastases, while higher mean attenuation is a significant predictor for small non-calcified pulmonary metastases (99). Studies have reported that radiological semantic features like lobulation, spiculation, subtlety, calcification, and texture were relevant along with automatically calculated image-based radiomics features for malignancy classification of pulmonary nodules, which is found to be in line with current clinical practice (83, 100). Inspired by the aforementioned work, in this study, both semantic radiological and clinical features of lung nodules will be combined with radiomics features to develop the proposed classification ML/DL predictive model for metastatic lung nodule detection. Harmonization of radiomics features is an important aspect to obtain standardization and reproducibility of the developed model. In this study, extracted radiomics features will be harmonized by the methods following the recent studies (101–104) as applicable.

ML/DL has achieved a series of satisfactory results in the field of medical imaging, and it also has made great progress in the detection and classification of lung nodules (14–20) and the prediction of nodule growth (105, 106) using ML/DL techniques in primary lung cancer. However, there has been a dearth of work reported on detection of lung metastases. There is no accurate non-invasive modality available to determine the malignant potential of a smaller (<5 mm) pulmonary nodule. Furthermore, functional imaging like 18F-FDG PET/CT scans are also not sensitive for lesions <1 cm. Because this ML/DL-based system will be trained using thousands of lung scans, it will be optimized to detect tiny malignant areas that specialists might overlook in the daily busy clinical routine. On successful completion of the project, the proposed model might be capable of assisting the radiologist to make a diagnostic decision combining the radiological screening with clinical data. The proposed classification model may shift the current clinical practice paradigms by utilizing novel non-invasive radiomics features of CT in combination with ML/DL techniques for characterizing indeterminate lung nodules in the patients with sarcomas. A working model for accurate detection of lung metastases may be effectively applicable for patients with different kinds of primary malignancy as well, especially those with predilection for lung metastases. Early detection of metastatic disease may help in planning personalized treatment protocol and may improve the OS rate.

The strength of the proposed study design is in analyzing an ambispective cohort with separate derivation and validation cohort to develop the proposed prediction and classification model. Moreover, combining radiological features and clinical features with various radiomics textural features may reduce the false-positive rate and help to produce a robust model. Furthermore, information about temporal changes in lung nodules during anti-cancer treatment will also be included into the feature map that will aid the prediction of the malignant potential of lung nodules. In addition, a number of ML and DL algorithms or a hybrid model combining both techniques will be implemented and tested to deliver an optimal performance.

There are few limitations in the proposed study. For demarcation of lung nodules in CT scans, an in-house-built segmentation tool has been used that has been tested on a limited number of 50 patients; however, the accuracy of the tool will be further evaluated in due course as larger data will be tested on the project. This project is multi-institutional and will be facilitated and carried out under AIIMS New Delhi and IIT Delhi in India. Retrospective and prospective datasets of patients with sarcoma will be collected from the institutional database at AIIMS New Delhi. In the future, a multi-institutional dataset may be considered for further improvement of the developed model. A comparison between limited ML and DL classification models will be performed for evaluating best performance; however, there might be a large number of ML/DL-based algorithms that need to be considered.

## 4 Ethics and dissemination

The ethical approval for the use of retrospective data and collection of prospective data of the patients with sarcomas has been obtained (IEC-234/09.04.2021, RP-03/2021) from the Institute Ethics Committee, AIIMS New Delhi, India. The details of data acquisition, processing, and sharing along with risks and benefits for participating in the study will be explained to all the patients before recruitment to the proposed project. Detailed patient information sheets written in English and Hindi (regional) languages have been reviewed and approved by the Institutional Ethics Committee.

The proposed study challenges and seeks to shift the current clinical practice paradigms by utilizing novel non-invasive radiomics features of CT combined with physiological symptoms and clinical biomarkers. As a result, misdiagnosis and false-positive rate might be reduced along with reduction in the total number of follow-up CT scans, which will subsequently reduce the time, cost, anxiety, and radiation exposure of the patients. Upon validation, the proposed model will be deployed in the local hospital settings and will be applied in the routine treatment protocol.

## Ethics statement

The studies involving human participants were reviewed and approved by the Institute Ethics Committee, All India Institute of Medical Sciences New Delhi, India. Written informed consent is obtained for the prospective cohort from the participants in case of adult subjects (18 years and older), from the legal guardian in case of those below 18 years, and additionally assent from children who are 8 years and above. The Institute Ethics Committee waives off consent for the use of the participants' data for the retrospective cohort.

## Author contributions

EBK, SG, KR, DK, SB, and AM conceptualized the study. Data curation and investigation were performed by EBK, SG, AS, SS, DDS, MS, and DK. Resources were provided by KR, DK, SB, and AM. Original manuscript was written by EBK. Reviewing and editing the manuscript were performed by EBK, SG, AS, SS, KR, DK, SB, and AM. All authors contributed to the article and read and approved the submitted version.
